# 9-*cis*-13,14-Dihydroretinoic Acid Is an Endogenous Retinoid Acting as RXR Ligand in Mice

**DOI:** 10.1371/journal.pgen.1005213

**Published:** 2015-06-01

**Authors:** Ralph Rühl, Agnieszka Krzyżosiak, Anna Niewiadomska-Cimicka, Natacha Rochel, Lajos Szeles, Belén Vaz, Marta Wietrzych-Schindler, Susana Álvarez, Monika Szklenar, Laszlo Nagy, Angel R. de Lera, Wojciech Krężel

**Affiliations:** 1 Department of Biochemistry and Molecular Biology, Faculty of Medicine, Debrecen, Hungary; 2 Paprika Bioanalytics BT, Debrecen, Hungary; 3 Institut de Génétique et de Biologie Moléculaire et Cellulaire (IGBMC), Illkirch, France; Inserm, U 964; 5 CNRS UMR 7104, Université de Strasbourg, Strasbourg, France; 5 DE-MTA “Lendület” Immunogenomics Research Group, University of Debrecen, Debrecen, Hungary; 6 Departamento de Química Orgánica and CINBIO, Facultad de Química, Universidade de Vigo, Vigo, Spain; 7 Instituto de Investigación Biomédica de Vigo (IBIV), Vigo, Spain; University of Aberdeen, UNITED STATES

## Abstract

The retinoid X receptors (RXRs) are ligand-activated transcription factors which heterodimerize with a number of nuclear hormone receptors, thereby controlling a variety of (patho)-physiological processes. Although synthetic RXR ligands are developed for the treatment of various diseases, endogenous ligand(s) for these receptors have not been conclusively identified. We show here that mice lacking cellular retinol binding protein (*Rbp1-/-*) display memory deficits reflecting compromised RXR signaling. Using HPLC-MS and chemical synthesis we identified in *Rbp1-/-* mice reduced levels of 9-*cis*-13,14-dihydroretinoic acid (9CDHRA), which acts as an RXR ligand since it binds and transactivates RXR in various assays. 9CDHRA rescues the *Rbp1-/-* phenotype similarly to a synthetic RXR ligand and displays similar transcriptional activity in cultured human dendritic cells. High endogenous levels of 9CDHRA in mice indicate physiological relevance of these data and that 9CDHRA acts as an endogenous RXR ligand.

## Author Summary

Daily nutrition, in addition to being a source of energy, contains micronutrients, a class of nutrients including vitamins which are essential for life and which act by orchestrating a vast number of developmental and physiological processes. During metabolism, micronutrients are frequently transformed into their bioactive forms. Nuclear hormone receptors are a family of proteins functioning as ligand-regulated transcription factors which can sense such bioactive molecules and translate those signals into transcriptional, adaptive responses. Retinoid X receptors occupy a central place in this signaling as they directly interact, and thereby control, activities of several nuclear hormone receptors. We report here the identification of a novel bioactive form of vitamin A, which is the first endogenous form of this vitamin capable to bind and activate retinoid X receptors. Accordingly, we show that this single molecule displays biological activity similar to synthetic agonists of retinoid X receptors and coordinates transcriptional activities of several nuclear receptor signaling pathways. Those findings may have immediate biomedical implications, as retinoid X receptors are implicated in the control of a number of physiological functions and their pathology.

## Introduction

Micronutrients such as vitamin A and polyunsaturated fatty acids are essential ingredients of mammalian diet and can act as bioactive molecules. Nuclear hormone receptors sense such molecular signals and accordingly regulate gene expression, thus functioning as ligand-controlled transcription factors. Retinoid X receptors (RXRs) occupy a central place in nuclear receptor signaling as obligatory heterodimerization partners for several of those receptors. RXR ligands can regulate the activity of only some heterodimers including for example LXR-RXR or PPAR-RXR, collectively called permissive heterodimers in opposition to non-permissive heterodimers, like RAR-RXR, which cannot be activated by RXR ligands alone [1,2]. Ligand-dependent modulation might be particularly relevant for the control of a wide range of physiological events. For example, among complex functions, working memory was shown sensitive to RXR ligand activities in mice [3], whereas at the cellular and molecular level, differentiation of monocyte-derived dendritic cells is one of the well characterized experimental models used to study the activities of RXR ligands [4,5]. Such ligands are also known to act as powerful inducers of apoptosis in cancer cells [6,7] or as modulators of lipid and glucose metabolism, which has stimulated their clinical development for the treatment of cancer and metabolic diseases [8]. Recent studies on antidepressant or neuro-regenerative activities of RXR specific agonists suggest also their utility for the treatment of some neuropsychiatric or neurodegenerative disorders [3,9,10].

In parallel to development and use of synthetic RXR ligands, several endogenous agonists have been proposed [11,12,13], but their physiological relevance remains questionable for different reasons. For example, 9*-cis*-retinoic acid (9CRA), an isomer of all-*trans*-retinoic acid (ATRA), was proposed and largely accepted as an endogenous RXR ligand [14,15]. However, although 9CRA can bind and activate RXRs at low concentrations, it was either undetectable [16,17,18,19,20,21] or was not present in sufficient concentrations [22] to enable RXR-mediated signaling in mammalian organisms. Docosahexaenoic acid (DHA), an alternative RXR ligand, was shown to bind and transactivate RXRs under pharmacological conditions [11,23,24], but in the physiological setting it can be detected in the brain mainly in esterified form contributing to e.g. structural components of the cell, while the pool of this fatty acid available for RXR activation remains too low [25,26]. Finally, phytanic acid [27,28] also suggested to bind RXR was not conclusively proven to be physiologically relevant.

In this study, we addressed the nature of known and novel endogenous retinoids and their role in RXR signaling *in vivo* by chemical, molecular and functional studies in distinct models.

## Results

**Fig 1:**
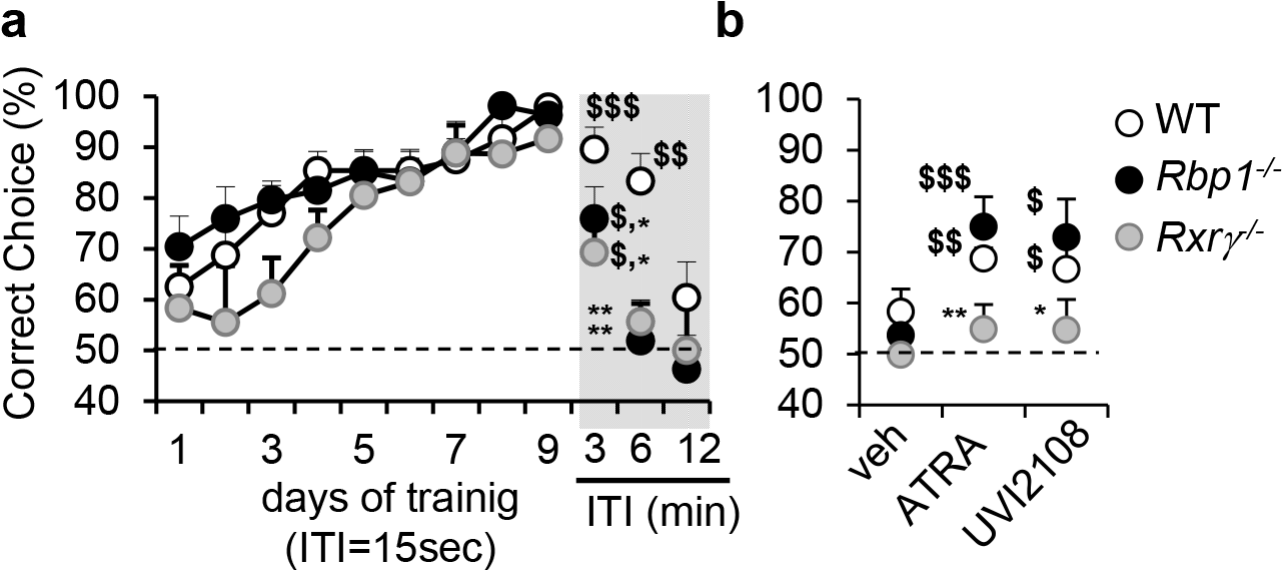
Compromised RXR signaling in *Rbp1* ^*-/-*^ mice. (a) *Rbp1* ^***-/-***^ (n = 8) and *Rxrγ* ^***-/-***^ (n = 7) mice acquired working memory DNMTP task similarly to WT (n = 8) mice (F[8,160] = 14, p<0.001; ANOVA on repeated measures) when trained with 15 sec inter-trial intervals (ITI), but showed forgetting when tested at 3, and 6 min ITIs, which was more rapid than 12 or 18 min in WT mice (indicated in the graph as 12 min ITI). (b) RXR agonist or ATRA improved working memory performance 6hrs after treatment in *Rbp1* ^***-/-***^ mice (n = 8) tested at 6 min ITI or WT mice (n = 8) at 12 or 18 min ITIs, but was inactive in *Rxrγ* ^***-/-***^ mice (n = 7) at ITI of 6 min. Compromised RXR signalling in *Rbp1* ^***-/-***^ was further supported in spontaneous alternation task, a distinct test for working memory (S1 Fig). Statistical differences identified with PLSD Fischer test were indicated as: *, p<0.05; **, p<0.01 as compared to WT controls in respective groups; $, p<0.05; $$, p<0.01; $$$, p<0.001 in comparison with 50% of chance level.

In order to search for endogenous retinoids which may act as RXR ligand(s), we first employed behavioral and pharmacological analyses sensitive to RXR signaling as a tool to identify animal models with reduced RXR signaling. In particular, we focused on spatial working memory previously reported as dependent on RXR and not RAR functions and more importantly also dependent on RXR ligand activities [3]. Using delayed non-match to place (DNMTP) task, we found that mice carrying a null mutation of cellular retinol binding protein I (RBP1), known for its role in retinoid metabolism [29], display memory deficits which phenocopy the effect of the loss of function of Rxrγ, a functionally predominant RXR in control of working memory (Fig 1A and ref. [3]). In particular, *Rbp1* ^*-/-*^ and *Rxrγ* ^*-/-*^ mice performed significantly worse when compared to wild type (WT) mice at 3 or 6 min inter-trial intervals (ITI) in DNMTP task, attaining chance level (complete forgetting) already at 6 min, whereas WT mice performed at chance level only at 12 or 18 min depending on individual (Fig 1A, see grey part of the left panel). These data suggest that RXR signaling is compromised in *Rbp1* ^*-/-*^ mice.

To challenge this hypothesis functionally, we took advantage of the sensitivity of working memory performance in delayed task to treatment with RXR agonists [3]. Activation of RXR signaling by the synthetic RXR agonist, UVI2108 (also known as SR11217 or BMS649) or by ATRA, which under pharmacological conditions is rapidly transformed *in vivo* to RXR agonist 9CRA[12,30], reversed memory deficits in *Rbp1* ^*-/-*^ mice, but remained ineffective in mice lacking RXRγ (Fig 1B). Working memory deficits were also observed in a distinct, rodent specific memory test of spontaneous alternation in the Y-maze (S1 Fig). Treatments with ATRA, UVI2108 and other RXR agonists, including DHA and methoprene acid, but not pan-RAR agonist TTNPB led to pro-mnemonic effects in *Rbp1* ^*-/-*^, but not *Rxrγ* ^*-/-*^ mice (S1 Fig), supporting the possibility of compromised RXR signaling due to reduced availability of RXR ligand(s) in *Rbp1* ^*-/-*^ mice. Accordingly, reduced expression of RXRγ could not explain behavioral deficits in *Rbp1* ^*-/-*^ mice. On the contrary, expression of RXRγ was clearly increased in *Rbp1* ^*-/-*^ striatum attaining level of 3.2 ± 0.6 in *Rbp1* ^*-/-*^ mice as compared to 1.2 ± 0.3 arbitrary RNA units (qRT-PCR).

**Fig 2:**
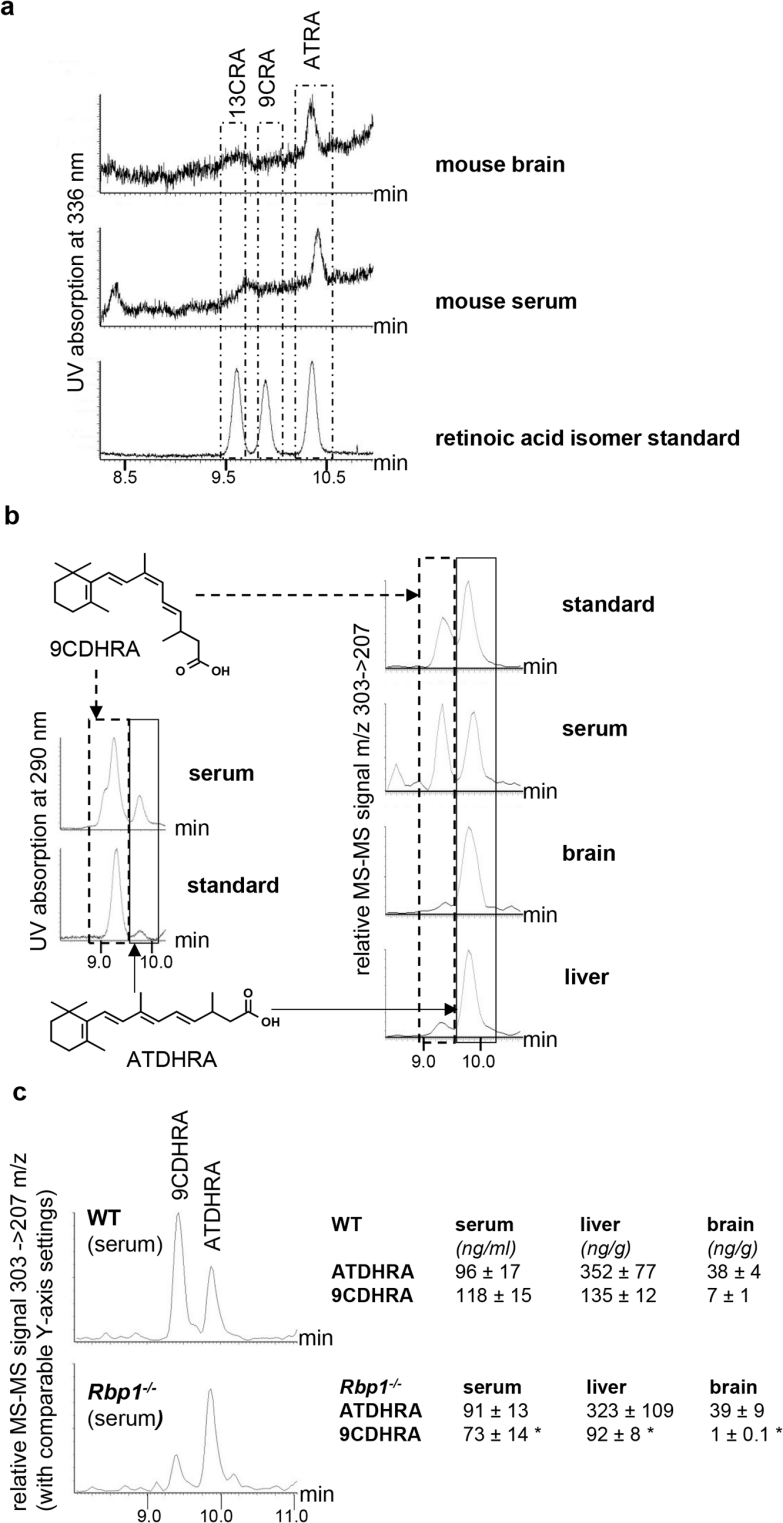
9CDHRA is present in mouse serum, brain and liver. (a) Elution profiles of WT mouse serum (n = 7) and brain (n = 3) samples in comparison with three retinoic acid standards (all-*trans* [ATRA], 13-*cis* [13CRA] and 9*-cis* retinoic acid [9CRA] with retention times 10.4, 9.6, and 9.9 min, respectively) allow identification of ATRA but not 9CRA. Boxes represent areas with comparable retention times to identify co-eluting peaks. (b) 9-*cis*-13,14-dihydroretinoic acid (9CDHRA; retention time 9.4 min highlighted by dotted-line box) is present in mouse serum (n = 7), brain (n = 3) and liver (n = 7) samples as determined by co-elution with a mixture of 9CDHRA and all-*trans*-13,14-dihydroretinoic acid (ATDHRA; retention time 9.9 min, highlighted by continuous-line box) standard solution and confirmed by MS-MS (303->207 m/z) and DAD (290 nm) detection. (c) Significant reduction of 9CDHRA, but not ATDHRA levels in serum, brain and liver of *Rbp1* ^***-/-***^ animals (n = 8, n = 3 for brain) as compared to WT mice (n = 8, n = 3 for brain). All the error bars represent S.E.M.

To evaluate RXR ligand availability in *Rbp1* ^*-/-*^ mice, we first addressed concentration of 9CRA in mouse brain and serum. Using a sensitive method of retinoic acid detection based on HPLC separation followed by highly specific DAD detection and destructive MS-MS [20], we clearly identified ATRA in serum (0.3 ± 0.1 ng/ml) and brain (0.6 ± 0.1 ng/g) samples from WT mice, whereas in the range of 9CRA elution no conclusive peak was identified indicating that 9CRA is absent or its levels were under our detection limit of 0.1 ng/g and thereby too low for sufficient RXR-activation in WT (Fig 2A) and *Rbp1* ^*-/-*^ animals. We then focused on dihydroretinoids described as novel endogenous retinoids [31,32]. Using stereo- and enantiocontrolled organic synthesis we obtained a series of dihydroretinoids, including all-*trans*-13,14-dihydroretinoic acid (ATDHRA) and its stereoisomer 9-*cis*-13,14-dihydroretinoic acid (9CDHRA; see Materials and methods for details of its synthesis), which we next used as reference molecules in HPLC-MS-MS analyses. Such analyses were focused on liver, as major site of Rbp1 expression, serum, through which retinoids are distributed to target organs, and brain, with discrete areas expressing Rbp1 [33]. We identified two major peaks, which co-eluted with standards of ATDHRA and 9CDHRA at UV specific absorption of 290 nm (Fig 2B, left panel). Such co-elution was also observed at dihydroretinoid-specific MS-MS settings (Fig 2B, right panel). Concentrations of 9CDHRA were high in serum samples attaining 118 ± 15 ng/ml (corresponding to ~4 x 10^-7^M), 135 ± 12 ng/g in mouse liver (corresponding to a concentration of ~7x10^-7^M) and relatively low (7 ± 1 ng/g, corresponding to ~2 x 10^-8^M) in brain. A direct comparison of these retinol metabolites in WT and littermate *Rbp1* ^*-/-*^ mice (Fig 2C) showed comparable concentration of ATDHRA in contrast to significantly decreased 9CDHRA levels in serum, liver and brain of *Rbp1* ^*-/-*^ mice. Importantly, whereas such decrease in serum may be at the origin of systemic reduction of RXR signaling, almost complete loss of 9CDHRA availability in brains of *Rbp1* ^*-/-*^ mice suggest more significant reduction of local RXR signalling in this organ. Furthermore, whole brain measures reflect most probably more dramatic changes of 9CDHRA levels in discrete brain areas expressing Rbp1 [33]. Unfortunately it is technically impossible to identify retinoid concentrations in these small areas, which can be only prompted by whole brain measures.

**Fig 3:**
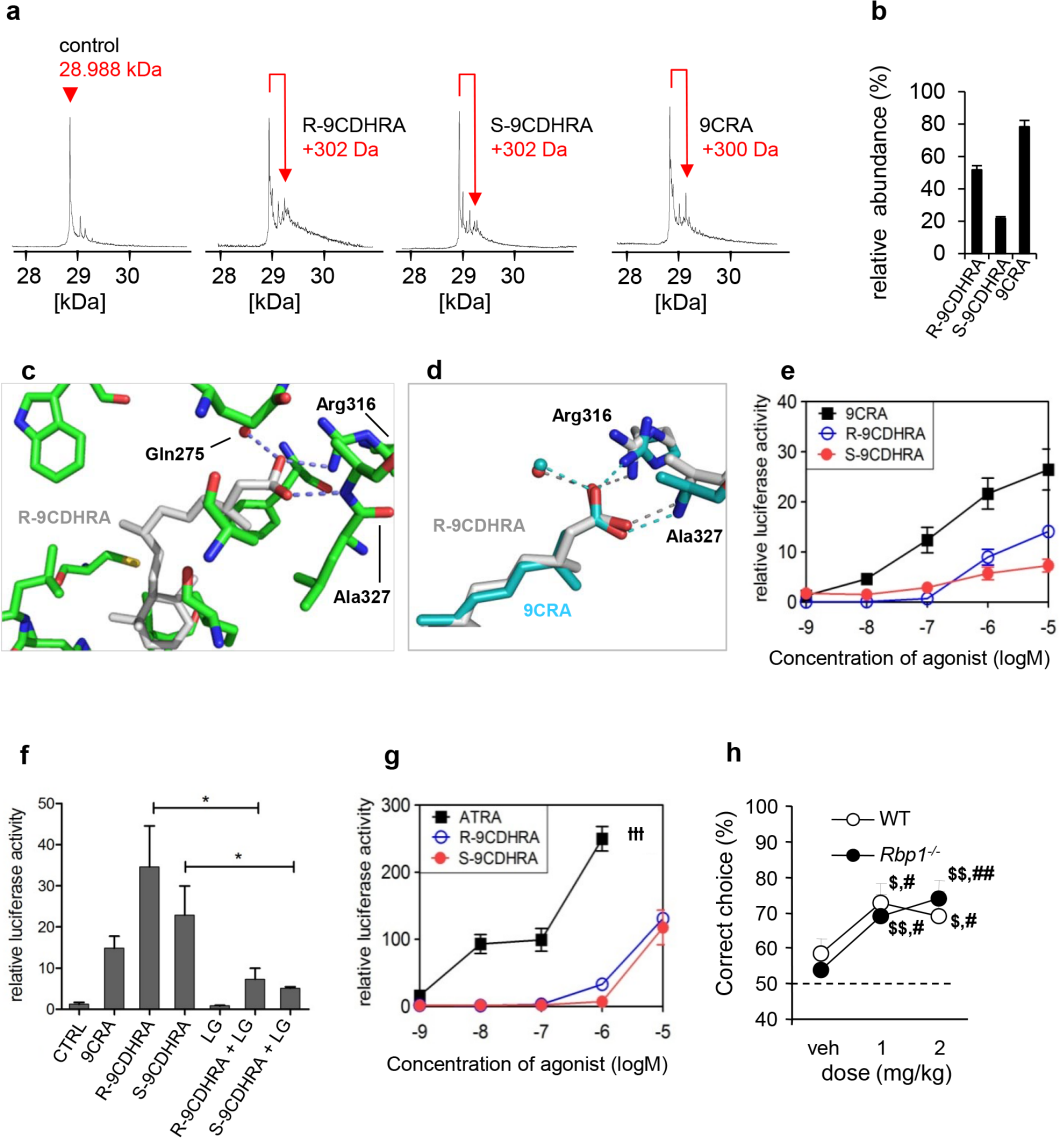
9CDHRA binds and transactivates RXR *in vitro*, and displays RXR agonist-like activities *in vivo*. (a) ESI mass spectra of hRXRα LBD protein after incubation with a 5-fold molar excess of 9CRA, R-9CDHRA and S-9CDHRA. (b) Distribution plot of the percentage of bound hRXRα. (c) Close-up view showing the ligand-binding pocket of RXRα bound to R-9CDHRA (in grey). Residues in close contacts (<3.5 Å) are labelled. Dashed lines indicate hydrogen bonds and the red sphere a water molecule. (d) Superposition of the RXRalpha ligand-binding pocket bound to R-9CDHRA (grey) and 9CRA (cyan, PDB code: 1XDK). Superposition was made on the protein. Hydrogen bonds with R-9CDHRA or 9CRA are shown by grey and cyan dashed lines, respectively. Water molecules are shown by spheres (red for RXR-R-9CDHRA and cyan for RXR-9CRA). (e) Transcriptional activation of RXRα by R-9CDHRA and S-9CDHRA in comparison to 9CRA (10^***–5***^–10^***-9***^M) in RXR-reporter COS1 cells. (f) 9CRA (10^***-7***^M) and both 9CDHRA enantiomers (10^***-5***^M) induced RXRα-mediated signaling. RXR-antagonist LG101208 (LG; 10^***-6***^M) diminished activity of both 9CDHRA enantiomers (10^***-5***^M). (g) Transcriptional activation of RAR-RXR heterodimers by R- and S-9CDHRA in comparison to ATRA in RARα-RXRα-reporter COS1 cells. (h) Increasing doses of R-9CDHRA reversed working memory deficits in *Rbp1* ^***-/-***^ mice and showed pro-mnemonic activity in WT mice (n = 8/group) in DNMTP task when tested at minimal ITI, at which mice performed at chance level (50%) and which was 6min for the *Rbp1-/-* or 12min for WT mice. ttt: ATRA at concentration 10^***–5***^ M was cytotoxic. *, p<0.05. #, p<0.05; ##, p<0.01 as compared to vehicle treatment in the same group; $, p<0.05; $$, p<0.01; one group student t-test for comparison with performance at chance level of 50%. All the error bars represent S.E.M.

Direct evidence for 9CDHRA binding to RXR was given by electrospray ionisation mass spectrometry (ESI-MS) performed in non-denaturing conditions with purified RXR ligand binding domains (LBD). In order to evaluate relative affinities of R- and S-enantiomers and 9CDHRA for RXR LBD, titration experiments were monitored by ESI-MS. As shown in Fig 3A and 3B, all retinoids bind to hRXRα LBD used in these studies as model RXR LBD due to high conservation of LBD structure among all RXRs. Analyses of peak amplitude revealed that R-9CDHRA has approximately 30% lower affinity than 9CRA, but about 65% higher affinity than S-9CDHRA. Quantitative binding affinities to RXRa LBD obtained by fluorescence quenching assay (S2 Fig) are equal to 90 ± 20 nM for R-9CDHRA and 20 ± 10 nM for 9CRA, and fall in the range of published Kd [34] indicating that 9CDHRA binds RXRs with high affinity at concentrations which are physiologically relevant. Since 9CRA can bind to RARs we also tested affinity of 9CDHRA for all three RAR isotypes. ESI-MS experiments performed with hRARα, β and γ LBDs (S3A and S3B Fig) revealed that 9CDHRA (R and S) bind to all RAR LBD isotypes.

To provide structural evidence of the binding of R-9CDHRA to RXR, the hRXRα LBD was crystallized in complex with R-9CDHRA and a 13-residue peptide comprising the nuclear receptor-binding surface NR2 of NCoA2. Note that the residues lining the ligand binding pocket are strictly conserved between RXRα and RXRγ and that the conclusions drawn for RXRα will also be valid for RXRγ. The structure refined at 1.8 Å resolution (S1 Table) revealed the canonical active agonist conformation common to all previously reported agonist-bound nuclear receptor LBDs with 12 or 13 α-helices organized in a three-layered sandwich (S3C and S3D Fig). R-9CDHRA adopts a similar binding mode as 9CRA [35,36] including interactions of the carboxylate group of the ligand with Arg316 (H5), and hydrogen bonds with the amide group of Ala327 in the beta turn (Fig 3C and 3D). The number of contacts is similar between the two ligands although some interactions are weaker in the case of R-9CDHRA compared to 9CRA as for example the interactions with Leu436 (4.0 Å instead of 3.6 Å for 9CRA), Arg316 (2.7 Å instead of 2.3 Å) or Trp305 (4.3 Å instead of 3.5 Å) that account for the weaker binding of R-9CDHRA. In silico comparison of S- and R-9CDHRA binding mode in RXRα LBP revealed that the opposite configuration at C13 leading to slightly different side chain conformation of S-9CDHRA may underlay its lower affinity to RXR (S3E Fig), further supported by the lower relative binding affinity measured by ESI-MS.

The relevance of R-9CDHRA and S-9CDHRA receptor binding for transcriptional activities of RXRs was tested in COS1 reporter cell lines transfected with a RXRα expression vector (Fig 3E–3G). In agreement with previous reports, 9CRA induced transcription of reporter gene at concentrations starting from 10^-9^M, whereas R-9CDHRA or S-9CDHRA displayed similar activity to 9CRA, although at concentrations higher than 10^-7^M. Importantly, the activities of R- and S-9CDHRA at 10^-5^M were prevented by co-treatment with an RXR-antagonist LG101208 at 10^-6^M (Fig 3F). 9CDHRAs also activated RAR-RXR signaling in COS1 model reporter cells (transfected with RARα and RXRα expression vectors) starting at 10^-6^M (Fig 3G). Considering that all RXR isotypes share the same structure of their ligand binding pockets, the present data obtained with the RXRα isotype indicate that 9CDHRA may efficiently bind to all RXRs and induce their transcriptional activities at concentrations found in physiological conditions.

Behavioral analyses revealed that the 9CDHRA modulation of RXR functions is also relevant *in vivo*. Accordingly, acute treatment with R-9CDHRA improved memory performance of *Rbp1* ^*-/-*^ mice as compared to vehicle treatment or chance level of 50% when tested in DNMTP task at ITI of 6 min (Fig 3H). R-9CDHRA treatments also raised performance of WT mice when tested at long ITIs of 12 or 18 min, at which time the corresponding WT mice treated with vehicle performed at chance level. Such treatment did not improve performance of *Rxrγ* ^*-/-*^ mice (57 ± 7% of correct choices) indicating RXR specificity of 9CDHRA effects, which is further supported by similar effects of 9CDHRA and UVI2108 treatments (compare Fig 3H and 3L).

**Fig 4:**
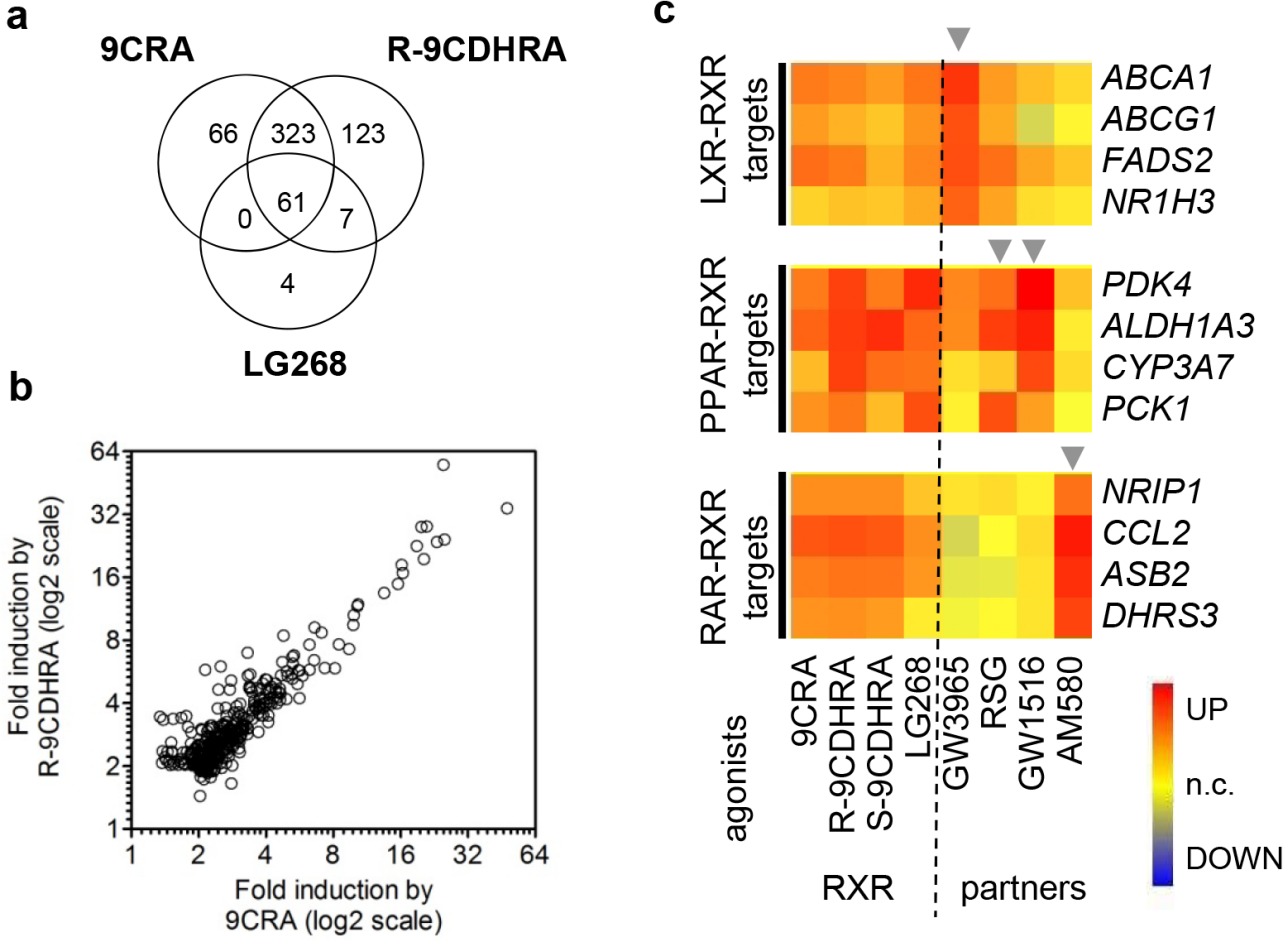
Molecular evidence for 9CDHRA selective activation of RXRs. (a) Significant overlap between global transcriptional changes induced by R-9CDHRA (10^***-5***^M), 9CRA (10^***-6***^M) or a synthetic RXR agonist (LG268; 10^***-7***^M) revealed by DNA microarray analyses in differentiating monocyte-derived human dendritic cells. (b) Scatter plot comparison of fold-changes for transcripts altered by 9CRA and R-9CDHRA treatments. (c) S- and R-9CDHRA, similarly to 9CRA and/or LG268 (see “RXR” columns), induced the expression of genes (see rows) identified previously as direct transcriptional targets of LXR-RXR, PPAR-RXR and RAR-RXR. Corresponding transcripts were also induced by agonists of respective RXR-heterodimer partners (see “partners” column and arrowheads): GW3965 (LXRα/β; 10^***-6***^M), RSG (PPARγ; 10^***-6***^M), GW1516 (PPARδ; 10^***-6***^M) and AM580 (RAR; 10^***-7***^M).

In order to identify 9CDHRA specificity for induction of RXR-dependent transcriptional activity at the transcriptomic level and its capacity to activate permissive heterodimers, we took advantage of human differentiating monocyte-derived dendritic cell cultures, a well characterized *in vitro* model for studies of signaling through RXR and its heterodimers [4,5]. The gene expression changes induced by R-9CDHRA, S-9CDHRA, other RXR ligands or ligands for RXR partners, revealed that R-9CDHRA and 9CRA regulate approximately the same number of transcripts (518 and 450, respectively; Fig 4). Importantly, 384 transcripts were similarly regulated by both agonists (Fig 4A and 4B), which corresponded to 85% of all transcripts regulated by 9CRA. Within this set, a group of 61 transcripts was also regulated by LG268, a synthetic RXR specific ligand used in our analysis as a reference in previous studies of this model [4,5]. Remarkably, none of the transcripts were regulated solely by 9CRA and LG268, and not by 9CDHRA, indicating that 9CDHRA induces similar gene expression changes as 9CRA.

We also investigated the capacity of 9CDHRA for activating permissive heterodimers, e.g. LXRα/γ-RXR, PPARγ -RXR, and PPARδ-RXR. As expected, we found that R-9CDHRA, similarly to 9CRA and LG268, could induce the expression of many genes, which are known as direct targets of RXR permissive heterodimers. Accordingly these genes were also regulated by LXR or PPAR specific ligands (Fig 4C). To address whether 9CDHRA also activates RAR-RXR target genes we compared the effect of RXR ligands and AM580, a synthetic RARα selective ligand. Typically genes induced by AM580 were not induced by any other agonist of permissive partners (Fig 4C), but were also induced by 9CDHRA or 9CRA. Collectively, gene expression profiling indicated that R- and S-9CDHRAs display RXR agonist activity, but can also activate RARs, acting thus with similar selectivity to 9CRA.

## Discussion

Although RXRs occupy central position in signaling of several nuclear hormone receptors acting as their heterodimerisation partner, endogenous ligand(s) of RXRs, its metabolic pathway and physiological functions were not conclusively determined. We found that *Rbp1* ^*-/-*^ displayed a phenotype suggestive of reduced RXR signaling, which could not be attributed to the reduced levels of RXR expression or of 9CRA, the potential endogenous RXR ligand which we and others failed to detect [16,17,18,19,20] in wild type animals. Using chemical approaches of HPLC-MS and organic synthesis we identified 9CDHRA, a novel endogenous retinoid, the concentrations of which were significantly reduced in serum, liver and brain of *Rbp1* ^*-/-*^ mice. Several lines of evidence indicate that 9CDHRA treatment activates RXRs *in vitro* and *in vivo* at physiologically relevant concentrations, suggesting that it acts as an endogenous RXR agonist.

We report herein that RBP1 modulates animal behavior by control of the availability of an RXR ligand. Accordingly, mice carrying null mutation of RBP1 display working memory deficits, the hallmark of deficient signalling through RXRγ, a functionally predominant RXR in the control of working memory in adult mice [3,37]. Reduced expression of RXRγ do not account for these changes, suggesting compromised availability of RXR ligand. This unique model enabled us to search for the endogenous RXR ligand(s). As our initial analyses failed to detect 9CRA in wild type and *Rbp1* ^*-/-*^ mice, we turned our attention to dihydroretinoids proposed recently as a novel group of bioactive, endogenous retinoids [31]. Using HPLC-MS-MS conditions specific for detection of dihydroretinoic acids, including 13,14-dihydroretinoic acids, and aided by organic synthesis we detected the presence of ATDHRA and 9CDHRA in mouse serum, liver and brain in WT mice and *Rbp1* ^*-/-*^ mice. Serum and liver concentration of 9CDHRA were particularly high, ranging from 4 to 7x10^-7^M, and much lower in whole-brain extracts in WT animals. Nevertheless they were significantly reduced in all corresponding samples of *Rbp1* ^*-/-*^ mice.

Reduced serum availability of 9CDHRA in *Rbp1* ^*-/-*^ mice may result from reduced synthesis of 9CDHRA in the liver, the main site of RBP1 expression [29]. Because levels of ATDHRA were comparable in the serum, liver and brain of WT and *Rbp1* ^*-/-*^ mice, reduced levels of 9CDHRA in *Rbp1* ^*-/-*^ mice indicate that RBP1 plays an important role specifically in generation of different forms of 9-*cis*-retinoids as previously suggested [38]. Thus, 9CDHRA, similarly to ATRA and 1,25-dihydroxy-vitamin D3 could act in endocrine and paracrine manner as a lipid hormone of nutritional origin being distributed in the serum but also synthetized locally in specific organs [39,40]. In consequence, reduced systemic levels of 9CDHRA may synergize with local reduction of its synthesis in specific brain areas of *Rbp1* ^*-/-*^ mice leading to compromised RXR activities and mnemonic deficits. In favor of this hypothesis, systemic administration of R-9CDHRA, a 9CDHRA enantiomer obtained by stereoselective chemical synthesis, normalized working memory deficits in *Rbp1* ^*-/-*^ mice. That such effects are mediated by RXRs may be suggested by absence of promnemonic effects of 9CDHRA and other RXR ligands in mice carrying null mutation of RXRγ, a functionally predominant RXR in the control of these brain functions [3].

Direct evidence of 9CDHRA binding to RXRs is provided by electrospray ionisation mass spectrometry (ESI-MS) performed in non-denaturing conditions using purified RXR LBD and fluorescence quenching assay. In particular, R-9CDHRA binds RXR LBD with affinity close to that of 9CRA as indicated by respective Kd values of 90 ± 20 nM for 9CDHRA and 20 ± 10 nM for 9CRA. Such data are supported by the crystal structure of the complex of R-9CDHRA with RXR LBD, in which R-9CDHRA adopts the canonical active agonist conformation and the carboxylate interacts with Arg316. Importantly, the R-9CDHRA enantiomer efficiently induces RXR transcriptional activity in reporter cell assays at physiologically relevant concentrations below 10^-6^M, which can be prevented by co-administration of RXR pan-antagonist LG101208. Although S-9CDHRA displays lower affinity to bind RXR LBD in ESI-MS, most probably due to the inverse positioning of the C20-carbon atom, it is also active in the transactivation of RXR in vitro with threshold concentration between 10^–7^ and 10^-6^M.

Further relevance of 9CDHRA for the activation of RXRs *in vitro* was indicated by the regulation of transcriptional targets of LXR-RXR or PPAR-RXR permissive heterodimers, which are known to be sensitive to pharmacological activation of RXR as well as its nuclear receptor partners. Such activation was demonstrated in human dendritic cells cultured *in vitro*, a well-established model for analyses of RXR signalling [4,5]. Importantly, almost all (68 out of 72) transcripts regulated by LG268 (RXR-specific agonist) were also regulated by 9CDHRA. Such a result obtained in a course of transcriptomics study was very similar to the data obtained for 9CRA, which controlled 61 out 72 LG268 transcriptional targets, indicating the extremely high capacity of 9CDHRA and 9CRA to control RXR transcriptional targets. Such genes correspond most probably to permissive heterodimers, and indirect targets of liganded RXR and their regulation provide evidence that 9CDHRA can control RXR signalling also in human cells. High degree of overlap between transcriptional activities of 9CRA and 9CDHRA, which goes beyond the activation of RXR-specific transcripts, reflects their capacity to bind and transactivate also RARs. That 9CRA and 9CDHRA act as a mixed RXR and RAR agonists is supported by about 80% overlap in transcriptional changes induced by R-9CDHRA (or S-9CDHRA) and 9CRA.

Whereas 13,14-dihydroretinol was detected by Moise and colleagues [31] as a hepatic retinol saturase (RETSAT) metabolite in the mammalian organism, other dihydroretinoids or their precursors were also identified in other vertebrates [41,42] and also non-vertebrates [43]. Besides RETSAT-mediated retinol metabolism as the major potential pathway for endogenous 9CDHRA synthesis, also apo-carotenoids and carotenoids may serve as substrates for dehydrogenation via RETSAT or other saturases, followed by the synthesis of dihydroretinoic acids [44,45].

This metabolic pathway may be phylogenetically ancient, as RXR orthologs from several non-vertebrate species including mollusk [46], primitive chordate like amphioxus [47] and some primitive insects like *Tribolium* [48] or *Locusta migratoria* [49,50] have also a potential to bind RXR ligands. In addition, *Locusta migratora* ultraspiracle (a fly RXR ortholog), displayed higher affinity to bind 9CRA than human RXR [49], raising the possibility that 9CDHRA could also activate the USP pathway and be an ancestral RXR ligand. Based on our data, it is tempting to suggest that in addition to the active ligands originating from vitamin A1 and vitamin A2, 9CDHRA and its nutritional precursors may represent a third novel distinct pathway of vitamin A metabolism and signaling, which evolved specifically to control RXR activity.

In summary we have characterized 9CDHRA as the first endogenous and physiologically relevant retinoid that acts as RXR ligand in mammals. Reduced memory functions due to compromised RXR-mediated signaling in *Rbp1* ^*-/-*^ mice result from lower brain levels of 9CDHRA, reflecting reduced serum transport and local brain synthesis of 9CDHRA in *Rbp1* ^*-/-*^ mice. Future determination of the metabolic pathways involved in 9CDHRA synthesis and signaling and its tissue specificity will be important for further understanding of the functional relevance of 9CDHRA to animal physiology, pathology and evolution.

## Materials and Methods

### Animals

*Rbp1* ^*-/-*^ and *Rxrγ* ^*-/-*^ mutants as well as wild type (WT) control mice were raised on a mixed genetic background (50% C57BL/6J and 50% 129SvEms/j; bred for more than 10 generations) from heterozygous crosses as described [29,51], and tested at the age of 3 months for chemical analyses and 3–6 months for behavioral analyses. All mice were housed in groups of 4–5 mice per cage in a 7am-7pm light/dark cycle in individually ventilated cages (Techniplast, Italy). Food (standard chow diet, D04 from SAFE, France) and water were freely available. All experiments were carried out in accordance with the European Community Council Directives of 24 November 1986 (86/609/EEC) and in compliance with the guidelines of CNRS and the French Agricultural and Forestry Ministry (decree 87848).

### Behavioral procedures

All behavioral tests were carried out in the Institute Clinique de la Souris (http://www.ics-mci.fr/) according to standard operating procedures. To study working memory, behaviorally naïve groups of mice were tested in the DNMTP in the T-maze according to a protocol previously described [52] with modifications to facilitate pharmacological tests [3]. Spontaneous alternation was evaluated in the Y-maze apparatus according to the protocol described in detail in the S1 Text.

### Analytical procedures

Serum and tissue samples for chemical analyses were collected during the light phase of the light/dark cycle between 3-4pm, around 9–10 hours after the last major food intake, which in our lighting conditions takes place around 6-7am. High performance liquid chromatography mass spectrometry–mass spectrometry (HPLC-MS-MS) analyses were performed under dark yellow/amber light using previously validated protocol [20]. For the detection of 13,14-dihydroretinoic acid MS-MS settings were 303 -> 207 m/z using the same dwell time and collision energy comparable to the MS-MS specific settings of retinoic acids. Quantification was performed as previously described [20]. For details of sample preparation see S1 Text.

### Reporter cell lines

COS1 cells were maintained in DMEM medium with 10% FBS, 5% L-glutamine, 1% penicillin streptomycin in 24-well plates and transfections were carried out in triplicates. Cells were transfected with equal amounts of relevant plasmids including Gal-RXRα-LBD for RXR-reporter line or Gal-RARα-LBD and Gal-RXRα-LBD for RAR-RXR reporter line, a reporter plasmid (luciferase MH100-TKLuc reporter construct with GAL-binding site [53] and beta-galactosidase (for transfection efficiency calculation). For details of transfection and measurements see the S1 Text.

### Binding assays

cDNAs encoding hRXRα LBD (223–462), hRARα LBD (153–421), hRARβ LBD (169–414) and hRARγ LBD (178–423) were cloned into the pET28b vector to generate N-terminal His-tag fusion proteins. Purification was carried out as previously described [54,55], including a metal affinity chromatography and a gel filtration. For details of sample preparation and ESI-MS and fluorescence quenching analyses see the S1 Text.

### Structure analysis of RXR-LBD in complex with R-9CDHRA

Details on crystallization, X-ray data collection and structure determination can be found in the S1 Text, S3 Fig and S1 Table. The coordinates and structure factors are deposited in the Protein Data Bank under the accession codes 4ZSH.

### Animal treatments

R-9CDHRA, UVI2108, ATRA (Sigma), DHA (Sigma), MA (Sigma) and TTNPB (Sigma), were dissolved in ethanol and DMSO, and then mixed with sunflower oil, so that the final solution contained 3% ethanol and 3% DMSO. Vehicle treatments consisted of 3% ethanol and 3% DMSO solution in sunflower oil. Treatments were administered by intraperitoneal injections at volume/weight ratio 3 ml/kg between 8-10am and 5–6 h before the test as previously validated [3].

### Human dendritic cell (DC) generation and DNA microarray analysis

The generation and transcriptional analysis of differentiating DCs were performed as described previously [5] and are detailed in the S1 Text. Microarray data were deposited into the Gene Expression Omnibus database under accession no. GSE48573.

### Statistical analysis

The comparisons of behavioral performance in Rbp1-/- and Rxrγ-/- mice were carried out using the protected least significant difference (PLSD) Fischer test. The pharmacological data for the treatments in WT and Rbp1-/- or Rxrγ-/- mice were analysed using 2-way analysis of variance (ANOVA)—with treatment and genotype as two independent factors and behavioral responses as dependent variables. The evolution of learning curves in WT, Rbp1-/- and Rxrγ-/- mice were done using ANOVA on repeated measures. Global and post-hoc statistical analyses were performed using student t-test for two-group comparisons. Significant differences are indicated in the corresponding figures.

### Chemical synthesis—Synthesis of dihydroretinoids

To confirm whether relative MS-MS signal detected at 303>207 m/z corresponds to 9CDHRA, the stereoselective synthesis of both enantiomers of 9-*cis*-13,14-dihydroretinoic acid was carried out following the previously described strategy based on a palladium-catalyzed Csp^2^-Csp^2^ Suzuki coupling [56]. Details of the stereocontrolled synthesis, purification and characterization of the (*R*)- and (*S*)-enantiomers of 9-*cis*-13,14-dihydroretinoic acid are provided in the S1 Text.

## Supporting Information

S1 Fig Compromised RXR signalling in Rbp1-/- mice. Working memory deficits observed in the spontaneous alternation task in the Y-maze could be normalised in Rbp1-/- mice, but not in Rxrγ -/- mice using all-*trans*-retinoic acid (ATRA; 5mg/kg), UVI2108 (1mg/kg), MA (5mg/kg), DHA (1mg/kg), but not TTNPB (5mg/kg) (n = 8-15/group). All compounds were applied 5–6 hours prior to testing and all mice were tested only one time in this task. Statistical differences revealed by PLSD Fisher test results were indicated: **, p<0.01; ***, p<0.001 as compared to WT animals in respective group.
(PDF)

S2 Fig Fluorescence quenching assay. (a) An example of fluorescence emission spectra for the binding of increasing amounts of 9CDHRA to RXRα LBD (1.25μxM). Curves 1–7 correspond to concentration of 0, 0.2, 0.45, 0.6, 1, 1.6, 1.2μM of 9CDHRA; (b) Plot of the uncorrected fluorescence of RXRα LBD in the presence of increasing amount of 9CDHRA. Analysis of the fluorescence quenching according to Cogan plot as described in [34] leads to a Kd value of 90 ± 20nM and N = 0.7 ± 0.03. N corresponds to number of binding sites.
(PDF)

S3 Fig Binding of 9CDHRA (R and S enantiomers) to RAR LBD isotypes in silico, and crystallography analyses of 9CDHRA binding to RXR. (a) ESI mass spectra of hRARα (top), hRARβ (middle) and hRARγ (bottom) LBDs protein after incubation with a 5-fold molar excess of ligands; (b) Distribution plot of the relative proportion of percent of bound/free protein for hRAR isotypes for R- and S-9CDHRA enantiomers and 9CRA. (c) Overall crystal structure of the RXRalpha LBD in complex with R-9CDHRA. (d) Experimental 2Fo-Fc electron density map of R-9CDHRA ligand contoured at 1sigma. (e) Modeling of the binding of S-9CDHRA in RXRalpha ligand binding pocket and superposition of S-9CDHRA (in cyan) and R-9CDHRA (in green) revealed that the side chain of S-9CDHRA adopts slightly different position due to the inverse configuration at C13, that results in a similar interaction of the carboxyl group but requires a side chain repositioning of Phe313 (Helix H5) that should result in a lower affinity to RXR. The S-9CDHRA generated with Marvin program was docked in the protein structure of the R-9CDHRA complex.
(PDF)

S1 Table Data collection and refinement statistics. ^a^ *R*_sym_ = 100 × S_h*j*_ |*I*_h*j*_—<*I*_h_>| / S_h*j*_ *I*_h*j*_, where *I*_h*j*_ is the *j*th measurement of the intensity of reflection h and <*I*_h_> is its mean value. ^b^ *R*_cryst_ = 100 × S||*F*_o_|–|*F*_c_|| / S|*F*_o_|, where |*F*_o_| and |*F*_c_| are the observed and calculated structure factor amplitudes, respectively. ^c^ Calculated using a random set containing 10% of observations that were not included throughout refinement [57].
(PDF)

S1 Text Additional information on materials and methods and detailed description of chemical synthesis procedures. (DOC)
