# Supplementary figures and images for: 9-cis-13,14-Dihydroretinoic Acid Is an Endogenous Retinoid Acting as RXR Ligand in Mice
Source: PLoS Genet. 2015 Jun 1;11(6):e1005213. doi: 10.1371/journal.pgen.1005213 (PMC4451509; doi:10.1371/journal.pgen.1005213)

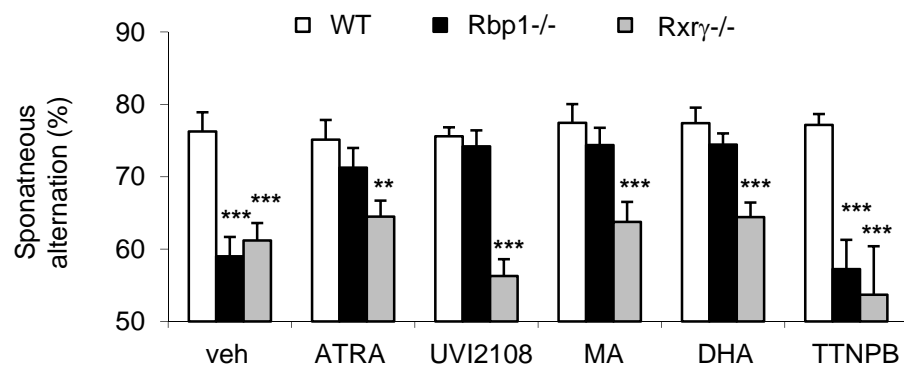

**Supporting Figure 1. Compromised RXR signalling in Rbp1<sup>-/-</sup> mice.**

Supplement: S1 Fig — Working memory deficits observed in the spontaneous alternation task in the Y-maze could be normalised in Rbp1-/- mice, but not in Rxrγ -/- mice using all-trans-retinoic acid (ATRA; 5mg/kg), UVI2108 (1mg/kg), MA (5mg/kg), DHA (1mg/kg), but not TTNPB (5mg/kg) (n = 8-15/group). All compounds were applied 5–6 hours prior to testing and all mice were tested only one time in this task. Statistical differences revealed by PLSD Fisher test results were indicated: **, p<0.01; ***, p<0.001 as compared to WT animals in respective group. (PDF) [file pgen.1005213.s001.pdf]

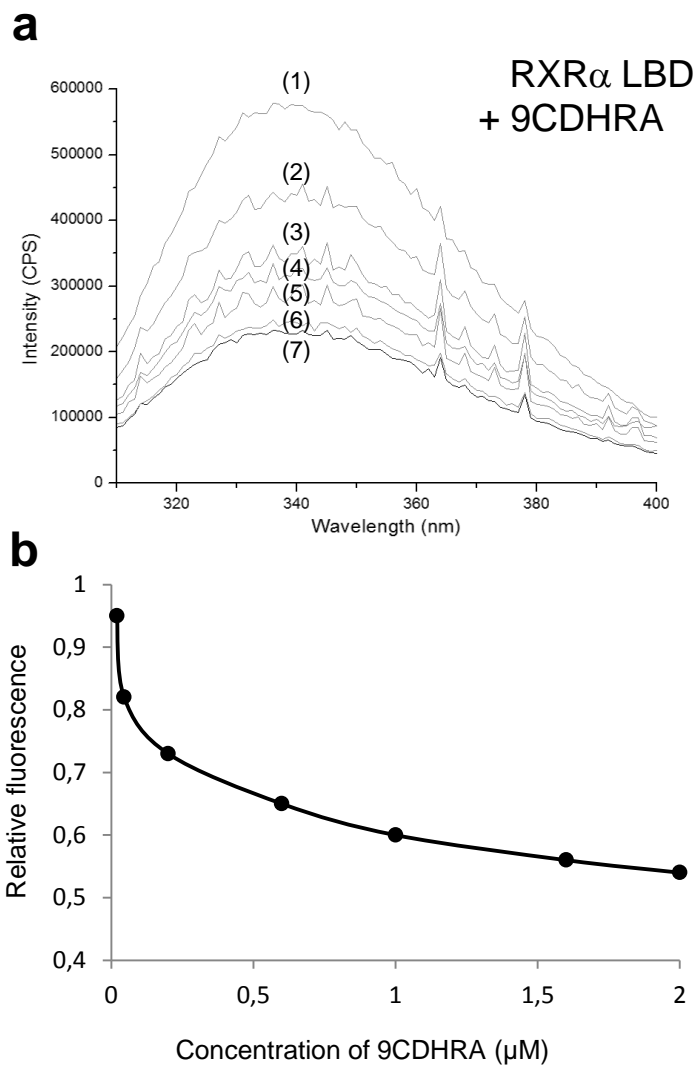

**Supporting Figure 2. Fluorescence quenching assay.**

Supplement: S2 Fig — (a) An example of fluorescence emission spectra for the binding of increasing amounts of 9CDHRA to RXRα LBD (1.25μxM). Curves 1–7 correspond to concentration of 0, 0.2, 0.45, 0.6, 1, 1.6, 1.2μM of 9CDHRA; (b) Plot of the uncorrected fluorescence of RXRα LBD in the presence of increasing amount of 9CDHRA. Analysis of the fluorescence quenching according to Cogan plot as described in [34] leads to a Kd value of 90 ± 20nM and N = 0.7 ± 0.03. N corresponds to number of binding sites. (PDF) [file pgen.1005213.s002.pdf]
