# Supplementary material for: 9-cis-13,14-Dihydroretinoic Acid Is an Endogenous Retinoid Acting as RXR Ligand in Mice
Source: PLoS Genet. 2015 Jun 1;11(6):e1005213. doi: 10.1371/journal.pgen.1005213 (PMC4451509; doi:10.1371/journal.pgen.1005213)
Supplement: S3 Fig — (a) ESI mass spectra of hRARα (top), hRARβ (middle) and hRARγ (bottom) LBDs protein after incubation with a 5-fold molar excess of ligands; (b) Distribution plot of the relative proportion of percent of bound/free protein for hRAR isotypes for R- and S-9CDHRA enantiomers and 9CRA. (c) Overall crystal structure of the RXRalpha LBD in complex with R-9CDHRA. (d) Experimental 2Fo-Fc electron density map of R-9CDHRA ligand contoured at 1sigma. (e) Modeling of the binding of S-9CDHRA in RXRalpha ligand binding pocket and superposition of S-9CDHRA (in cyan) and R-9CDHRA (in green) revealed that the side chain of S-9CDHRA adopts slightly different position due to the inverse configuration at C13, that results in a similar interaction of the carboxyl group but requires a side chain repositioning of Phe313 (Helix H5) that should result in a lower affinity to RXR. The S-9CDHRA generated with Marvin program was docked in the protein structure of the R-9CDHRA complex. (PDF) [file pgen.1005213.s003.pdf]

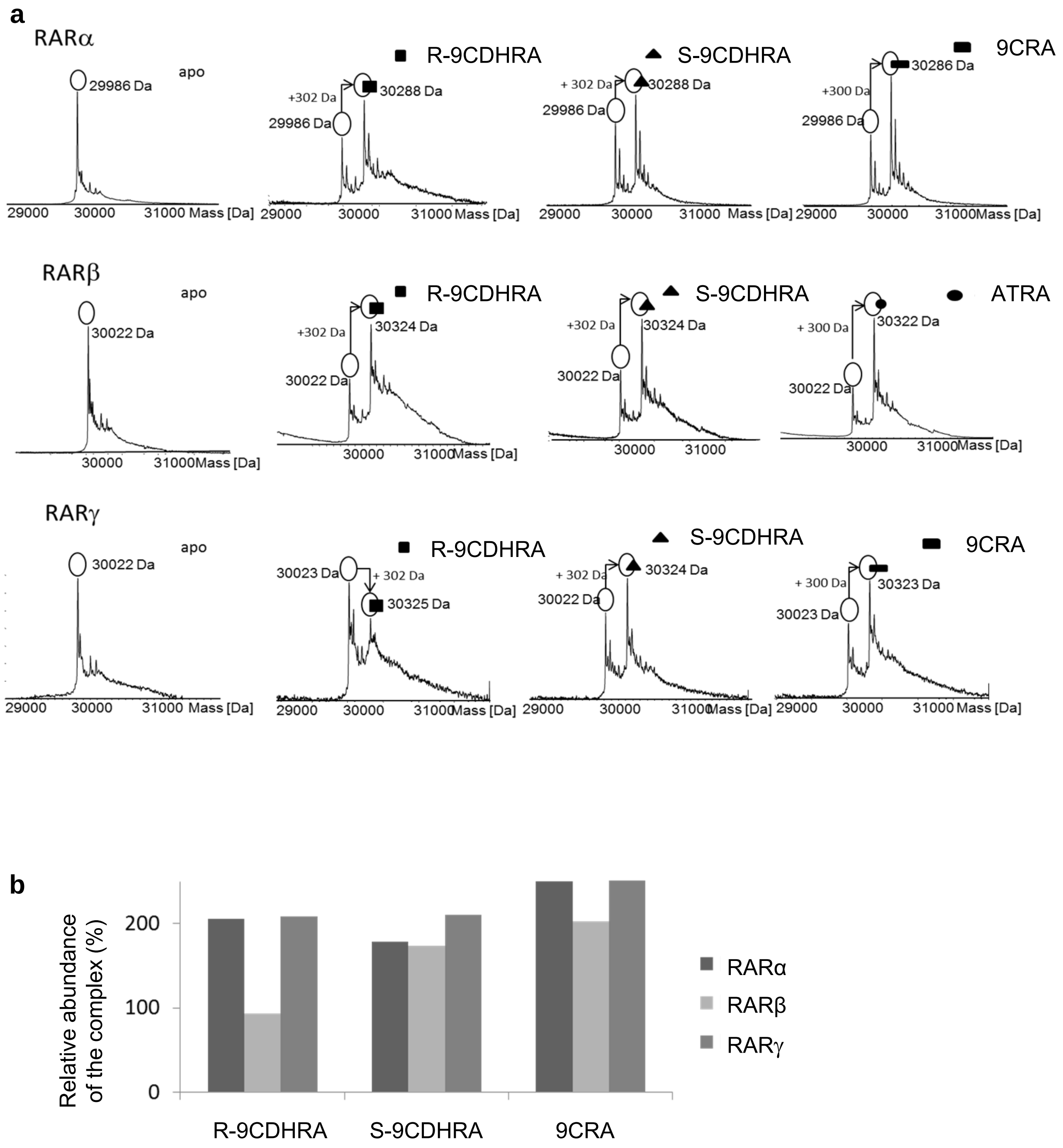

a
RARα
apo
29986 Da
R-9CDHRA
+302 Da
29986 Da
30288 Da
S-9CDHRA
+ 302 Da
29986 Da
30288 Da
9CRA
+300 Da
29986 Da
30286 Da
29000
30000
31000 Mass [Da]
RARβ
apo
30022 Da
R-9CDHRA
+302 Da
30022 Da
30324 Da
S-9CDHRA
+302 Da
30022 Da
30324 Da
ATRA
+ 300 Da
30022 Da
30322 Da
30000
31000 Mass [Da]
RARγ
apo
30022 Da
R-9CDHRA
30023 Da
+ 302 Da
30325 Da
S-9CDHRA
+ 302 Da
30022 Da
30324 Da
9CRA
+ 300 Da
30023 Da
30323 Da
29000
30000
31000 Mass [Da]
b
Relative abundance of the complex (%)
0
100
200
R-9CDHRA
S-9CDHRA
9CRA
RARα
RARβ
RARγ

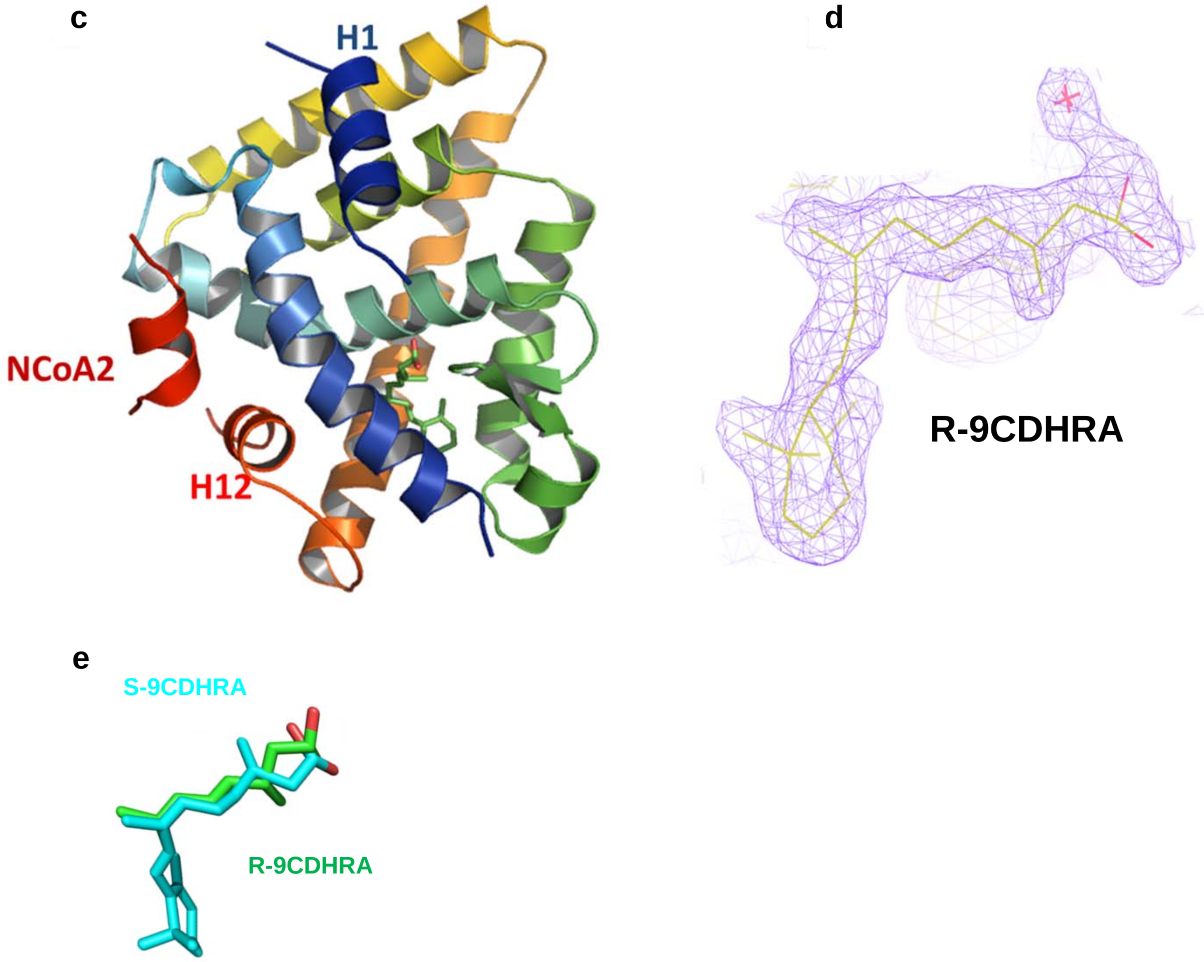

Supporting Figure 3 : Binding of 9CDHRA (R and S enantiomers) to RAR LBD isotypes in non-denaturing ESI-MS assays and crystallography, and in silico analyses of 9CDHRA binding to RXR.
