# Supplementary material for: 9-cis-13,14-Dihydroretinoic Acid Is an Endogenous Retinoid Acting as RXR Ligand in Mice
Source: PLoS Genet. 2015 Jun 1;11(6):e1005213. doi: 10.1371/journal.pgen.1005213 (PMC4451509; doi:10.1371/journal.pgen.1005213)
Supplement: S1 Table — a R sym = 100 × Shj |I hj—| / Shj I hj, where I hj is the jth measurement of the intensity of reflection h and is its mean value. b R cryst = 100 × S||F o|–|F c|| / S|F o|, where |F o| and |F c| are the observed and calculated structure factor amplitudes, respectively. c Calculated using a random set containing 10% of observations that were not included throughout refinement [57]. (PDF) [file pgen.1005213.s004.pdf]

**RXRα LBD/R-DHRA /NCoA2**	
Data processing	
Resolution (Å)	25-1.8 (1.86-1.80)
Crystal space group	$P4_32_12$
Cell parameters (Å)	*a* = *b* = 64.014; *c* = 112.066
Unique reflections	22407
Mean redundancy	7.9 (3.8)
R_{sym} (%)[a]	5.2 (48.1)
Completeness (%)	99.2 (97.2)
Mean I/σ	34.7(2.3)
Refinement	
Resolution (Å)	24-1.8
Number of non-hydrogen atoms	
RXR-LBD	1680
Coactivator peptide	98
Ligand	22
Water molecules	181
RMSD bond length (Å)	0.006
RMSD bond angles (˚)	1.065
R_{cryst} (%)[b]	17.9
R_{free} (%)[c]	22.1
Averaged *B* factor for non-hydrogen atoms (Å^2)	
RXR-LBD	37.6
Coactivator peptide	44.6
R-DHRA	31.2
Water	44.5

SupportingTable 1 **Data collection and refinement statistics.**
