# Supplementary material for: 9-cis-13,14-Dihydroretinoic Acid Is an Endogenous Retinoid Acting as RXR Ligand in Mice
Source: PLoS Genet. 2015 Jun 1;11(6):e1005213. doi: 10.1371/journal.pgen.1005213 (PMC4451509; doi:10.1371/journal.pgen.1005213)
Supplement: S1 Text — (DOC) [file pgen.1005213.s005.doc]

**TEXT S1**

**MATERIALS AND METHODS**

**Behavioral procedures**

***Delayed non-match to place (DNMTP):*** Behaviourally naïve groups of mice were tested in the DNMTP in the T-maze according to a protocol previously described [52], with modifications to facilitate pharmacological tests. Specifically, in the present protocol, animals were first trained for 9 consecutive days of with minimal inter-trial intervals (ITI) of about 15 sec, which was necessary to attain the criterion of 90% or more of correct choices during three consecutive days (days 7-9). After this period ITI was increased semi-randomly to 180, 360, 720 and for some WT mice also 1080 seconds so that each animal was tested 6 times with each interval during four consecutive days (days 10-13 indicated). From day 13 mice were tested only twice per week beginning with training session on day one (with minimal, 15 sec ITIs) and testing pharmacological treatments on the second day. Only one pharmacological treatment was tested every 7 days. To test pro-mnemonic activities of UVI2108 and R-9CDHRA we used a minimal ITI, at which mice performed at chance level (50%) and which was 6min for the Rbp1-/- or Rxrγ-/- groups and 12min for most WT mice with exception of two mice tested at 18min. Four mice (two wild types and one of each knockout line), were excluded from testing since their latency to choose the arm during the retention phase exceeded 3 min in more than one trial/day on three consecutive days (exclusion criterion).

***Y-maze spontaneous alternation:*** The Y-maze apparatus and the procedure were as previously described [52]. Briefly, each mouse was placed at the end of one arm and allowed to explore freely the apparatus for 5min, with the experimenter out of the animal’s sight. Spontaneous alternation performance (SAP) was assessed visually by scoring the pattern of entries into each arm and expressed as a percentage of total number of entries, discounting the first two visits. Total entries were scored as an index of ambulatory activity in the Y-maze, but none of tested mice, had to be excluded due to low locomotor performance (score below 9 entries).

**Analytical procedures:**

High performance liquid chromatography mass spectrometry (2695XE separation module; Waters, Hungary) –mass spectrometry (Micromass Quattro Ultima PT; Waters, Hungary) analyses were performed under dark yellow/amber light using previously validated protocol [20]. For the detection of 13,14-dihydroretinoic acid MS-MS setting were 303 -> 207 m/z using the same dwell time and collision energy comparable to MS-MS specific settings of retinoic acids. Quantification was performed comparable to RA quantification previously described in [20]. Accordingly, for sample preparation 100 mg of the material (if samples were under 100 mg, water was added up to the used standard weight: 100 mg) or 100 μl serum was diluted with a threefold volume of isopropanol, the tissues were minced by scissors, vortexed for 10 seconds, put in a ultra sonic bath for 5 minutes, shaken for 6 minutes and centrifuged at 13000 rpm in a Heraeus BIOFUGE Fresco at +4°C. After centrifugation, the supernatants were dried in an Eppendorf concentrator 5301 (Eppendorf, Germany) at 30°C. The dried extracts were resuspended with 60 μl of methanol, diluted with 40 μl of 60 mM aqueous ammonium acetate solution and transferred into the autosampler and subsequently analysed.

**Reporter cell lines:**

COS1 cells were maintained in DMEM medium with 10% FBS, 5% L-glutamine, 1% penicillin streptomycin in 24-well plates and transfections were carried out in triplicates. Cells were transfected with equal amounts of relevant plasmids including Gal-RXRα-LBD for RXR-reporter line or Gal-RARα-LBD and Gal-RXRα-LBD for RAR-RXR reporter line, a reporter plasmid (luciferase MH100-TKLuc reporter construct with GAL-binding site [53]) and beta-galactosidase (for transfection efficiency calculation). Transfection was carried out using PEI (Sigma) as transfection reagent. DMSO solution of each ligand was added 6h later and cells were incubated for 48h at 37°C. Luciferase activity was determined in the lysates of the cells using the Luciferase Assay Kit (Promega). Measurements were made with a Wallac Victor2 multilabel counter. The signal of each sample was normalized to β-galactosidase activity to take the transfection efficiency and cell viability into account.

**Binding assays**

cDNAs encoding hRXRαLBD (223-462), hRARαLBD (153-421), hRARβLBD (169-414) and hRARγ LBD (178-423) were cloned into pET28b vector to generate N-terminal His-tag fusion proteins. Purification was carried out as previously described [54,55], including a metal affinity chromatography and a gel filtration.

Prior to ESI-MS analysis, samples were desalted on Zeba Spin desalting columns (Pierce) in 150 mM ammonium acetate (pH 8.0). ESI-MS measurements were performed on an electrospray time-of-flight mass spectrometer (MicrOTOF, Bruker Daltonic, Germany). Purity and homogeneity of the proteins were verified by mass spectrometry in denaturing conditions (samples were diluted at 2 pmol/μl in a 1:1 water-acetonitrile mixture (v/v) acidified with 1% formic acid). The mass measurements of the noncovalent complexes were performed in ammonium acetate (200 mM; pH 8.0). Samples were diluted to 8 pmol/ml in the previous buffer and continuously infused into the ESI ion source at a flow rate of 3 ml/min through a Harvard syringe pump (Harvard Apparatus model 11). A careful optimization of the interface parameters was performed to obtain the best sensitivity and spectrum quality without affecting the noncovalent complexes stability. In particular, the capillary exit (CE) ranged from 60 to 150 V with a vacuum interface pressure of 2.3 mbar and was set to 80 V. For ligand-interaction analysis, ligands were added to the proteins in a 5 fold molar excess.

**Fluorescence quenching assay**

Fluorescence spectra were measured as previously described [34,58] using a Fluoromax-4 Horiba spectrophotometer. RXRa LBD was prepared as for ESI-MS analyses and incubated with different concentrations of 9CDHRA or 9CRA in Tris 10 mM, NaCl 100 mM buffer. Quenching of tryptophan fluorescence was monitored at 10°C using 5 nm of excitation and 5 nm of emission slit-width. The excitation wavelength was 295 nm and the emission spectra were measured between 260 and 450 nm. Corrections for inner filter effect were performed and data were analyzed by Cogan plot as described [34].

**Structure analysis of RXR-LBD in complex with R-9CDHRA**

Crystals of the complex of hRXRα LBD/9C13,14DHRA (R) and TIF-2 peptide were obtained at 17°C by vapor diffusion in hanging drops by mixing of 0.5 µl of the protein solution and 0.5 µl of reservoir solution which contains 50 mM calcium acetate and 18% PEG3350. The crystals were mounted in fiber loops and flash-cooled in liquid nitrogen after cryoprotection with the reservoir solution plus 5% ethylene glycol. Data collection from the frozen crystal was performed at 100 K on the beamline ID29 at the ESRF (Grenoble, France). The crystal belongs to the tetragonal space group P4_3_2_1_2, with one monomer per asymmetric unit. The data were integrated and scaled with HKL2000 [59] (statistics in Supplementary Table 1). The structure was solved and refined as described [54]. Refinement involved iterative cycles of manual building and refinement calculations. Anisotropic scaling, a bulk solvent correction and TLS restraints were used for the refinement. Individual atomic B factors were refined isotropically. Solvent molecules were then placed according to unassigned peaks in the electron density map. For additional information see also Supplementary Fig. 2 and Supplementary Table 1.

**Animal treatments**

R-9CDHRA, UVI2108, ATRA (Sigma), DHA (Sigma), MA (Sigma) and TTNPB (Sigma), were dissolved in ethanol and DMSO, and then mixed with sunflower oil, so that the final solution contained 3% ethanol and 3% DMSO. Vehicle treatments consisted of 3% ethanol and 3% DMSO solution in sunflower oil. Treatments were administered by intraperitoneal injections at volume/weight ratio 3 ml/kg between 8-10am and 5-6 h before the test as previously validated [3].

**Human dendritic cell (DC) generation and DNA microarray analysis**

The generation and analysis of differentiating DCs were performed as described previously [5] with minor modifications. Briefly, human monocytes were isolated from healthy volunteer's buffy coat, obtained with a Regional Ethical Board permit from the Regional Blood Bank and isolated by Ficoll gradient centrifugation followed by immunomagnetic cell separation, and were cultured in the presence of GM-CSF and IL-4. Differentiating DCs were stimulated 18 h after plating by various agonists for 12h. The ligands were used in the following concentrations: 10 μM R-9CDHRA, 10 μM S-9CDHRA, 1 μM 9cisRA, 100 nM LG100268, 1 μM GW3965, 1 μM Rosiglitazone (RSG), 1 μM GW1516, 100 nM AM580. Experiments were performed in biological triplicates. Samples were processed and hybridized to Human Genome U133 Plus 2.0 Arrays. Data were analysed using to GeneSpring 12.6.1 software. In detail, normalization was performed using RMA summarization algorithm. Significantly regulated genes were identified using a two-fold change cut-off (only transcripts with FC>2 were included) and moderated t-test with the Benjamini-Hochberg procedure for multiple test correction (FDR<0.05). Microarray data were deposited into the Gene Expression Omnibus database under accession no. GSE48573.

**Statistical analysis**

The comparisons of behavioral performance in Rbp1-/- and Rxrγ-/- mice were carried out using the protected least significant difference (PLSD) Fischer test. The pharmacological data for the treatments in WT and Rbp1-/- or Rxrγ-/- mice were analysed using 2-way analysis of variance (ANOVA) – with treatment and genotype as two independent factors and behavioral responses as dependent variables. The evolution of learning curves in WT, Rbp1-/- and Rxrγ-/- mice were done using ANOVA on repeated measures. Global and post-hoc statistical analyses were performed using student t-test for two-group comparisons or for three-group comparisons the PLSD Fischer test was used. Significant differences are indicated in the corresponding Figures.

**Chemical synthesis**

The preparation of (*R*)-9-*cis*-13,14-dihydroretinoic acid (*R*)-**1** was based on the Suzuki coupling of enantiopure trienyliodide **3** and boronic acid **2** (Scheme 1). The synthesis of **3** started with (*Z*)-stannyldienol **5** [60,61] which was transformed into the benzothiazolyl allyl sulfide **6** by Mitsunobu reaction with the corresponding thiol and subsequently oxidized to sulfone **7** with H_2_O_2_ and a peroxymolybdate (VI) reagent [62] at -10 ºC. The Julia-Kocienski olefination [63,64] was performed using a slight excess of base (NaHMDS, 1.15 equiv.) and 1.7 equivalents of enantiopure aldehyde (*R*)-**8** [65,66]. As anticipated from previous findings on the stereochemical outcome of the reactions of allylsulfones and aldehydes,[67,68] the newly formed olefin of trienyl ester (*R*)-**9** is of *Z*-geometry (which was confirmed by NOE experiments). Treatment of the precursor stannane with a solution of iodine in CH_2_Cl_2_ produced the iodide (*R*)-**3** via Sn-I exchange and iodine-promoted isomerization of the 9*Z*,11*Z* diene to the desired 9*Z*,11*E* geometric isomer (as confirmed by NOE experiments).

**Scheme 1**. Reagents and conditions: (a) PPh_3_, BTSH, DIAD, CH_2_Cl_2_, 2h (98%). (b) (NH_4_)_6_Mo_7_O_24_ **.** 4H_2_O, 30% H_2_O_2_, EtOH, -10 ºC, 17h (66%). (c) NaHMDS, THF, -78 ºC, 30 min (93%). (d) I_2_, CH_2_Cl_2_, 25 ºC, 30 min. (e) Pd(PPh_3_)_4_, 10% aq. TlOH, THF, 25 ºC, 3h (42%). (f) 2M KOH, MeOH, 80 ºC, 45 min (84%).

The Suzuki reaction of freshly prepared boronic acid **2** and dienyl iodide (*R*)-**3** using Pd(PPh_3_)_4_ as catalyst and 10% aq. TlOH as base in THF at ambient temperature, followed by immediate work-up afforded ethyl (*R*)-9-*cis*-13,14-dihydroretinoate (*R*)-**4** in 78% yield. Saponification of *R*-**4** provided in 84% yield the desired carboxylic acid (*R*)-**1** without detectable loss of stereochemical integrity.

Following the general scheme, enantiomer (*S*)-**1** was also prepared with similar efficiency.

**Preparation and Characterization of Compounds - General procedures**

Solvents were dried according to published methods and distilled before use. All other reagents were commercial compounds of the highest purity available. Unless otherwise indicated all reactions were carried out under argon atmosphere, and those not involving aqueous reagents were carried out in oven-dried glassware. Analytical thin layer chromatography (TLC) was performed on aluminium plates with Merck Kieselgel 60F254 and visualized by UV irradiation (254 nm) or by staining with an ethanolic solution of phosphomolibdic acid. Flash column chromatography was carried out using Merck Kieselgel 60 (230-400 mesh) under pressure. Electron impact (EI) mass spectra were obtained on a Hewlett-Packard HP59970 instrument operating at 70 eV. Alternatively an APEX III FT-ICR MS (Bruker Daltonics, Billerica, MA), equipped with a 7T actively shielded magnet was used and ions were generated using an Apollo API electrospray ionization (ESI) source, with a voltage between 1800 and 2200 V (to optimize ionization efficiency) applied to the needle, and a counter voltage of 450 V applied to the capillary. For ESI spectra samples were prepared by adding a spray solution of 70:29.9:0.1 (v/v/v) CH_3_OH/water/formic acid to a solution of the sample at a v/v ratio of 1 to 5% to give the best signal-to-noise ratio. High Resolution mass spectra were taken on a VG Autospec instrument. ^1^H NMR spectra were recorded in C_6_D_6_ and acetone-d_6_ at ambient temperature on a Bruker AMX-400 spectrometer at 400 MHz with residual protic solvent as the internal reference.

**(2*Z*,4*E*)-1-(Benzothiazol-2-yl)sulfanyl-5-(tri-*n*-butylstannyl)-3-methylpenta-2,4-diene (6).**

A solution of (2*Z*,4*E*)-3-methyl-5-(tributylstannyl)penta-2,4-dien-1-ol (1.0 g, 2.58 mmol), 2-mercaptobenzothiazol (0.65 g, 3.87 mmol) and PPh_3_ (1.10 g, 4.21 mmol) in THF (14 mL) was stirred for 5 min at 0 ºC. A solution of DIAD (0.77 mL, 3.87 mmol) in THF (5 mL) was added dropwise and the mixture was stirred for 30 min at 25 ºC. The solvent was removed and the residue was purified by column chromatography (C18-silicagel, CH_3_CN) to afford 1.11 g (78%) of a colorless oil identified as (2*Z*,4*E*)-1-(Benzothiazol-2-yl)sulfanyl-5-(tri-*n*-butylstannyl)-3-methylpenta-2,4-diene **6**. **^1^H NMR** (400 MHz, C_6_D_6_) δ 8.04 (d, *J* = 8.2 Hz, 1H, CH), 7.46 (d, *J* = 19.2 Hz, ^3^*J*_SnH_ = 71.2 Hz, 1H, CH), 7.34 (d, *J* = 8.0 Hz, 1H, CH), 7.21 (ddd, *J* = 8.3, 7.3, 1.2 Hz, 1H, CH), 7.01 (ddd, *J* = 8.3, 7.4, 1.1 Hz, 1H, CH), 6.66 (d, *J* = 19.2 Hz, ^2^*J*_SnH_ = 71.2 Hz, 1H, CH), 5.65 (t, *J* = 8.1 Hz, 1H, CH), 4.37 (d, *J* = 8.2 Hz, 2H, CH_2_), 1.86 (s, 3H, CH_3_), 1.8-1.6 (m, 6H, CH_2_), 1.5-1.4 (m, 6H, CH_2_), 1.1-1.0 (m, 15H, CH_3_ + CH_2_) ppm. **^13^C NMR** (100 MHz, C_6_D_6_) δ 166.4 (s), 153.7 (s), 142.6 (d), 138.6 (s), 135.7 (s), 132.0 (d), 125.9 (d), 124.1 (d), 122.1 (d), 121.7 (d), 121.0 (d), 30.2 (t), 29.3 (t, 3x), 27.6 (t, 3x), 19.8 (q), 13.8 (q, 3x), 9.7 (t, 3x) ppm; **IR** (NaCl) ν 2955 (s, C-H), 2923 (s, C-H), 2870 (m, C-H), 2850 (m, C-H), 1460 (s), 1427 (s) cm^-1^. **HRMS** (ESI^+^) *m/z* calcd for C_25_H_40_NS_2_^120^Sn: 538.1620; found: 538.1621.

**(2*Z*,4*E*)-1-(Benzothiazol-2-yl)sulfonyl-5-(tri-*n*-butylstannyl)-3-methylpenta-2,4-diene (7).** To a solution of (2*Z*,4*E*)-1-(benzothiazol-2-yl)sulfanyl-5-(tri-*n*-butylstannyl)-3-methylpenta-2,4-diene **6** (0.48 g, 0.89 mmol) in EtOH (9 mL), at -10 °C, was added a solution of (NH_4_)_6_Mo_7_O_24_·4H_2_O, (0.44 g, 0.36 mmol) in aqueous hydrogen peroxide (35%, 7.7 mL, 89.1 mmol). After stirring for 17 h at -10 ºC, the mixture was quenched with H_2_O and extracted with Et_2_O (3x). The combined organic layers were washed with brine (3x) and dried (Na_2_SO_4_), and the solvent was removed. The residue was purified by chromatography (C18-silicagel, MeOH) to afford 0.33 g (66%) of a colorless oil identified as (2*Z*,4*E*)-1-(benzothiazol-2-yl)sulfonyl-5-(tri-*n*-butylstannyl)-3-methylpenta-2,4-diene **7**. **^1^H NMR** (400 MHz, C_6_D_6_) δ 8.10 (d, *J* = 8.2 Hz, 1H, CH), 7.18 (d, *J* = 19.2 Hz, 1H, CH), 7.16 (t, *J* = 7.5 Hz,1H, CH), 7.12 (t, *J* = 8.1 Hz, 1H, CH), 6.98 (t, *J* = 7.7 Hz, 1H, CH), 6.59 (d, *J* = 19.2 Hz, ^2^*J*_SnH_ = 68.2 Hz, 1H, CH), 5.43 (t, *J* = 8.0 Hz, 1H, CH), 4.32 (d, *J* = 8.1 Hz, 2H, CH_2_), 1.8-1.6 (m, 9H, CH_3_ + CH_2_), 1.6-1.4 (m, 6H, CH_2_), 1.1-1.0 (m, 15H, CH_3_ + CH_2_) ppm. **^13^C NMR** (100 MHz, C_6_D_6_) δ 167.1 (s), 152.9 (s), 143.1 (s), 141.7 (d), 136.9 (s), 134.4 (d), 127.3 (d), 127.1 (d), 125.0 (d), 122.1 (d), 112.0 (d), 53.3 (t), 29.3 (t, 3x), 27.5 (t, 3x), 20.0 (q), 13.8 (q, 3x), 9.6 (t, 3x) ppm; **IR** (NaCl) ν 2955 (s, C-H), 2923 (s, C-H), 2850 (m, C-H), 1467 (m), 1333 (s), 1151 (s) cm^-1^. **HRMS** (ESI^+^) *m/z* calcd for C_25_H_39_NNaO_2_S_2_^120^Sn: 592.1338; found: 592.1334. **UV** (MeOH) λ_max_ 239 nm.

**(3*S*,4*Z*,6*Z*,8*E*)-Ethyl 3,7-Dimethyl-9-(tri-*n*-butylstannyl)nona-4,6,8-trienoate ((*S*)-9).** A cooled (-78 ºC) solution of (2*Z*,4*E*)-1-(benzothiazol-2-yl)sulfonyl-5-(tri-*n*-butylstannyl)-3-methylpenta-2,4-diene (0.115 g, 0.20 mmol) in THF (9 mL) was treated with NaHMDS (0.23 mL, 1M in THF, 0.23 mmol). After stirring for 30 min at this temperature, a solution of (*S*)-ethyl 3-methyl-4-oxobutanoate (0.044 g, 0.30 mmol) in THF (4.5 mL) was added and the resulting mixture was stirred for 1h at -78 ºC and 3h letting the temperature reach to rt. Et_2_O and water were added at low temperature and the mixture was warmed up to room temperature. It was then diluted with Et_2_O and the layers were separated. The aqueous layer was extracted with Et_2_O (3x), the combined organic layers were dried (Na_2_SO_4_) and the solvent was removed. The residue was purified by column chromatography (C-18 silica gel, MeOH) to afford 0.94 g (93%) of a pale yellow oil identified as (3*S*,4*Z*,6*Z*,8*E*)-ethyl 3,7-dimethyl-9-(tri-*n*-butylstannyl)nona-4,6,8-trienoate (*S*)-**9**. **[α]^28^_D_** +11.5º (*c* 1.22, MeOH). **^1^H NMR** (400 MHz, C_6_D_6_) δ 7.60 (d, *J* = 19.1 Hz, ^3^*J*_SnH_ = 65.1 Hz, 1H, CH), 6.85 (t, *J* = 11.4 Hz, 1H, CH), 6.64 (d, *J* = 19.2 Hz, ^2^*J*_SnH_ = 71.9 Hz 1H, CH), 6.58 (d, *J* = 11.9 Hz, 1H, CH), 5.31 (t, *J* = 10.4 Hz, 1H, CH), 4.03 (q, *J* = 7.1 Hz, 2H, CH_2_), 3.5-3.3 (m, 1H, CH), 2.4-2.2 (m, 2H, CH_2_), 2.02 (s, 3H, CH_3_), 1.8-1.6 (m, 6H, CH_2_), 1.6-1.4 (m, 6H, CH_2_), 1.2-0.9 (m, 18H, CH_3_ + CH_2_) ppm. **^13^C NMR** (100 MHz, C_6_D_6_) δ 171.3 (s), 143.2(d), 135.8 (d), 135.4 (s), 130.7 (d), 123.8 (d), 122.9 (d), 59.8 (t), 41.8 (t), 29.3 (t, 3x), 29.2 (d), 27.5 (t, 3x), 20.7 (q), 20.3 (q), 14.0 (q), 13.7 (q, 3x), 9.6 (t, 3x) ppm. **IR** (NaCl) ν 2957 (s, C-H), 2924 (s, C-H), 2871 (m, C-H), 2851 (m, C-H), 1737 (s, C=O), 1459 (m), 1160 (m) cm^-1^. **HRMS** (ESI^+^) *m/z* calcd for C_25_H_46_NaO_2_^120^Sn: 521.2416; found: 521.2408. **UV** (MeOH) λ_max_ 285 nm.

**(3*R*,4*Z*,6*Z*,8*E*)-Ethyl 3,7-Dimethyl-9-(tri-*n*-butylstannyl)nona-4,6,8-trienoate ((*R*)-9). [α]^29^_D_** -10.3º (*c* 1.25, MeOH).

**(3*R*,4*E*,6*Z*,8*E*)-Ethyl 9-Iodo-3,7-dimethylnona-4,6,8-trienoate ((*R*)-3)**. To a solution of (3*R*,4*Z*,6*Z*,8*E*)-ethyl 3,7-dimethyl-9-(tri-*n*-butylstannyl)nona-4,6,8-trienoate **(*R*)-9** (0.060 g, 0.121 mmol) in CH_2_Cl_2_ (5.3 mL) was added dropwise iodine (0.046 g, 0.182 mmol) in CH_2_Cl_2_ (2.8 mL) and the resulting mixture was stirred for 30 min at 25 ºC. A saturated Na_2_S_2_O_3_ solution was added and the reaction mixture was extracted with Et_2_O (3x), the combined organic layers were dried (Na_2_SO_4_) and the solvent was removed. The residue was purified by column chromatography (silica gel, 97:3 hexane/Et_3_N) to afford 0.037 g (92%) of a pale yellow oil identified as (3*R*,4*E*,6*Z*,8*E*)-ethyl 9-iodo-3,7-dimethylnona-4,6,8-trienoate (*R*)-**3**. **[α]^24^_D_** +14.8º (*c* 0.74, MeOH). **^1^H NMR** (400 MHz, C_6_D_6_) δ 7.65 (d, *J* = 14.5 Hz, 1H, CH), 6.28 (dd, *J* = 14.9, 11.3 Hz, 1H, CH), 6.05 (d, *J* = 14.5 Hz, 1H, CH), 5.64 (d, *J* = 11.1 Hz, 1H, CH), 5.44 (dd, *J* = 15.0, 7.8 Hz, 1H, CH), 3.95 (q, *J* = 7.1 Hz, 2H, CH_2_), 2.64 (dt, *J* = 14.1, 7.0 Hz, 1H, CH), 2.15 (dd, *J* = 14.9, 7.1 Hz, 1H, CH), 2.06 (dd, *J* = 14.9, 7.3 Hz, 1H, CH), 0.97 (t, *J* = 7.2 Hz, 3H, CH_3_), 0.88 (d, *J* = 6.8 Hz, 3H, CH_3_) ppm. **^13^C NMR** (100 MHz, C_6_D_6_) δ 171.9 (s), 142.7 (d), 140.4 (d), 132.7 (s), 130.9 (d), 124.6 (d), 78.5 (d), 60.5 (t), 41.8 (t), 34.5 (d), 20.4 (q), 19.9 (q), 14.7 (q) ppm. **IR** (NaCl) ν 2972 (m, C-H), 2932 (m, C-H), 1731 (s, C=O), 1666 (m), 1180 (m) cm^-1^. **HRMS** (ESI^+^) *m/z* calcd for C_13_H_19_INaO_2_: 357.0322; found: 357.0316. **UV** (MeOH) λ_max_ 275 nm.

**(3*S*,4*E*,6*Z*,8*E*)-Ethyl 9-Iodo-3,7-dimethylnona-4,6,8-trienoate ((*S*)-3). [α]^23^_D_** -16.7º (*c* 0.72, MeOH).

**(9*Z*,13*R*)-Ethyl 13,14-Didehydroretinoate ((*R*)-4).** To a solution of (3*R*,4*E*,6*Z*,8*E*)-ethyl 9-iodo-3,7-dimethylnona-4,6,8-trienoate (*R*)-**3** (0.036 g, 0.107 mmol) in THF (2.3 mL) was added Pd(PPh_3_)_4_ (0.013 g, 0.011 mmol). After 5 min at room temperature, 2,6,6-trimethylcyclohex-1-enylboronic acid **2** (0.027 g, 0.161 mmol) was added in one portion followed by TlOH (10% aqueous solution, 0.75 mL, 0.407 mmol). After stirring for 3 h at 25 ºC, Et_2_O was added and the reaction mixture was filtered through a short pad of Celite®. The filtrate was washed with NaHCO_3_ (sat) and the organic layer was dried (Na_2_SO_4_) and the solvent was removed. The residue was purified by column chromatography (silica gel, 97:3 hexane/Et_3_N) to afford 0.028 g (78%) of a pale yellow oil identified as (9*Z*,13*R*)-ethyl 13,14-didehydroretinoate (*R*)-**4**. **[α]^23^_D_** +14.2º (*c* 0.48, MeOH). **^1^H NMR** (400 MHz, C_6_D_6_) δ 6.90 (d, *J* = 16.0 Hz, 1H, CH), 6.71 (dd, *J* = 14.9, 11.2 Hz, 1H, CH), 6.28 (d, *J* = 16.0 Hz, 1H, CH), 5.96 (d, *J* = 11.1 Hz, 1H, CH), 5.51 (dd, *J* = 15.0, 7.8 Hz, 1H, CH), 3.94 (q, *J* = 7.2 Hz, 2H, CH_2_), 2.77 (dt, *J* = 14.1, 7.1 Hz, 1H, CH), 2.21 (dd, *J* = 14.8, 7.3 Hz, 1H, CH), 2.11 (dd, *J* = 14.8, 7.2 Hz, 1H, CH), 1.95 (t, *J* = 6.1 Hz, 2H, CH_2_), 1.90 (s, 3H), 1.79 (s, 3H), 1.66 – 1.52 (m, 2H), 1.52 – 1.41 (m, 2H), 1.11 (s, 6H), 0.95 (t, *J* = 7.1 Hz, 3H, CH_3_), 0.94 (d, *J* = 6.8 Hz, 3H, CH_3_) ppm. **^13^C NMR** (100 MHz, C_6_D_6_)  171.4 (s), 138.3 (s), 137.6 (d), 132.7 (s), 130.6 (d), 129.1 (d), 129.0 (s), 127.9 (d), 124.8 (d), 59.8 (t), 41.6 (t), 39.6 (t), 34.2 (s), 34.1 (d), 33.0 (t), 28.9 (q, 2x), 21.8 (q), 20.4 (q), 20.1 (q), 19.5 (t), 14.1 (q) ppm. **IR** (NaCl) ν 2961 (s, C-H), 2929 (s, C-H), 2866 (m, C-H), 1737 (s, C=O), 1455 (m), 1372 (m), 1167 (m) cm^-1^. **HRMS** (ESI^+^) *m/z* calcd for C_22_H_35_O_2_: 331.2632; found: 331.2625. **UV** (MeOH) λ_max_ 287 nm (ε = 20000 L·mol^-1^·cm^-1^).

**(9*Z*,13*S*)-Ethyl 13,14-didehydroretinoate ((*S*)-4). [α]^22^_D_** -15.5º (*c* 0.51, MeOH).

**(9*Z*,13*R*)-13,14-Didehydroretinoic Acid ((*R*)-1).** To a solution of (9*Z*,13*R*)-ethyl 13,14-didehydroretinoate (*R*)-**4** (0.023 g, 0.069 mmol) in MeOH (4.7 mL) was added KOH (2M aqueous solution, 1.1 mL, 2.27 mmol) and the reaction mixture was stirred for 45 min at 80 ºC. After letting the reaction cool down to room temperature, CH_2_Cl_2_ and brine were added and the layers were separated. The aqueous layer was washed with H_2_O (3x). The combined aqueous layers were acidified with HCl 10% and extracted with CH_2_Cl_2_ (3x). The combined organic layers were dried (Na_2_SO_4_) and the solvent was removed. The residue was purified by column chromatography (silica gel, gradient from 95:5 to 90:10 CH_2_Cl_2_/MeOH) to afford 0.017 g (84%) of a pale yellow oil identified as (9*Z*,13*R*)-13,14-didehydroretinoic acid (*R*)-**1**. **[α]^22^_D_** +7.1º (*c* 0.67, MeOH). **^1^H NMR** (400 MHz, acetone-d_6_): δ= 6.66 (d, *J* = 16.0 Hz, 1H), 6.60 (dd, *J* = 15.0, 11.2 Hz, 1H), 6.18 (d, *J* = 16.0 Hz, 1H), 5.93 (d, *J* = 11.1 Hz, 1H), 5.65 (dd, *J* = 15.0, 7.5 Hz, 1H), 2.73 (dt, *J* = 13.9, 7.0 Hz, 1H), 2.4 – 2.2 (m, 2H), 2.1 – 2.0 (m, 1H), 1.91 (s, 3H), 1.72 (s, 3H), 1.7 – 1.6 (m, 2H), 1.5 – 1.4 (m, 2H), 1.08 (d, *J* = 6.8 Hz, 3H), 1.03 (s, 6H) ppm. **^13^C NMR** (100 MHz, acetone-d_6_)  173.5 (s), 138.9 (s), 138.8 (d), 133.4 (s), 131.1 (d), 129.8 (s), 129.7 (d), 128.5 (d), 125.4 (d), 41.8 (t), 40.3 (t), 34.9 (s), 34.6 (d), 33.6 (t), 29.4 (q), 22.1 (q), 20.7 (q), 20.6 (q), 20.0 (t) ppm. **IR** (NaCl) ν 2957 (s, C-H), 2923 (s, C-H), 2855 (m, C-H), 1709 (s, C=O), 1446 (m), 1290 (m) cm^-1^. **HRMS** (ESI^+^) *m/z* calcd for C_20_H_31_O_2_: 303.2319; found: 303.2313. **UV** (MeOH) λ_max_ 289 nm (ε = 17600 L·mol^-1^·cm^-1^).

**(9*Z*,13*S*)-13,14-Didehydroretinoic Acid ((*S*)-1). [α]^24^_D_** -6.9º (*c* 0.26, MeOH).

**SUPPLEMENTARY REFERENCES**

58. Zhan Y, Du X, Chen H, Liu J, Zhao B, et al. (2008) Cytosporone B is an agonist for nuclear orphan receptor Nur77. Nat Chem Biol 4: 548-556.

59. Otwinowski Z, Minor W, editors (1997) Processing of X-ray Diffraction Data Collected in Oscillation Mode. Macromolecular Crystallography, part A ed. New York: Academic Press. 307-326 p.

60. Domínguez B, Pazos Y, de Lera AR (2000) Stereocontrolled Synthesis of 6-s-cis and 6-s-trans-Locked 9Z-Retinoids by Hydroxyl-Accelerated Stille Coupling of (Z)-Tri-n-Butylstannylbut-2-en-1-ol and Bicyclic Dienyl Triflates. J Org Chem 65: 5917-5925.

61. Pazos Y, de Lera AR (1999) Stereoselective Synthesis of 9-cis-Retinoic Acid Based on Stepwise or Convergent Suzuki Coupling Reactions. Tetrahedron Lett 40: 8287-8290.

62. Schultz HS, Freyermuth HB, Buc SR (1963) New Catalysts for the Oxidation of Sulfides to Sulfones with Hydrogen Peroxide. J Org Chem 28: 1140-1142.

63. Blakemore PR (2002) The modified Julia olefination: alkene synthesis via the condensation of metallated heteroarylalkylsulfones with carbonyl compounds. J Chem Soc Perkin Trans 1: 2563-2585.

64. Aïssa C (2009) Mechanistic Manifold and New Development of the Julia-Kocienski Reaction. Eur J Org Chem: 1831-1844.

65. Moise AR, Domínguez M, Alvarez S, Alvarez R, Schupp M, et al. (2008) Stereospecificity of Retinol Saturase:  Absolute Configuration, Synthesis, and Biological Evaluation of Dihydroretinoids. J Am Chem Soc 130: 1154-1155.

66. Leonard J, Mohialdin S, Reed D, Ryan G, Swain PA (1995) Stereoselective conjugate addition of organolithium and organocopper reagents to [delta]-oxygenated [alpha],[beta]-unsaturated carbonyl systems derived from glyceraldehyde acetonide. Tetrahedron 51: 12843-12858.

67. Sorg A, Brückner R (2005) Unexpected cis-Selectivity in (Sylvestre) Julia Olefinations with Bu3Sn-Containing Allyl Benzothiazolyl Sulfones: Stereoselective Synthesis of 1,3-Butadienyl- and 1,3,5-Hexatrienylstannanes. Synlett 2005: 289,293.

68. Vaz B, Alvarez R, Souto JA, de Lera AR (2005) γ-Allenyl Allyl Benzothiazole Sulfonyl Anions Undergo cis-Selective (Sylvestre) Julia Olefinations. Synlett 2005: 294,298.
